# Fabrication of Polymer/Graphene Biocomposites for Tissue Engineering

**DOI:** 10.3390/polym14051038

**Published:** 2022-03-04

**Authors:** João Meneses, Tom van de Kemp, Raquel Costa-Almeida, Rúben Pereira, Fernão D. Magalhães, Miguel Castilho, Artur M. Pinto

**Affiliations:** 1 LEPABE, Faculdade de Engenharia, Universidade do Porto, Rua Roberto Frias, 4200-465 Porto, Portugal; joao.meneses@inl.int (J.M.); t.vandekemp@students.uu.nl (T.v.d.K.); fdmagalh@fe.up.pt (F.D.M.); 2 International Iberian Nanotechnology Laboratory, 4715-330 Braga, Portugal; 3 i3S—Instituto de Investigação e Inovacão em Saúde, Universidade do Porto, 4200-135 Porto, Portugal; raquelccalmeida@gmail.com (R.C.-A.); ruben.pereira@ineb.up.pt (R.P.); 4 INEB—Instituto de Engenharia Biomédica, Universidade do Porto, 4200-135 Porto, Portugal; 5 Department of Orthopedics, University Medical Center Utrecht, 3584 CX Utrecht, The Netherlands; m.dias.castilho@tue.nl; 6 ICBAS—Instituto de Ciências Biomédicas Abel Salazar, Universidade do Porto, 4050-313 Porto, Portugal; 7 Department of Biomedical Engineering, Eindhoven University of Technology, 5600 MB Eindhoven, The Netherlands

**Keywords:** additive manufacturing, graphene-based materials, electrospinning, synthetic polymers, tissue engineering

## Abstract

Graphene-based materials (GBM) are considered one of the 21st century’s most promising materials, as they are incredibly light, strong, thin and have remarkable electrical and thermal properties. As a result, over the past decade, their combination with a diverse range of synthetic polymers has been explored in tissue engineering (TE) and regenerative medicine (RM). In addition, a wide range of methods for fabricating polymer/GBM scaffolds have been reported. This review provides an overview of the most recent advances in polymer/GBM composite development and fabrication, focusing on methods such as electrospinning and additive manufacturing (AM). As a future outlook, this work stresses the need for more in vivo studies to validate polymer/GBM composite scaffolds for TE applications, and gives insight on their fabrication by state-of-the-art processing technologies.

## 1. Introduction

Globally, around 310 million major surgical procedures are performed annually [1]. Most of these involve the repair or replacement of damaged tissues and/or organs due to disease or injury. Currently, most treatments include autografts and allografts. Despite promising outcomes, both approaches have important limitations. On one hand, the amount of available donor tissue, as well as the need for a second injury site, which results in additional trauma to the patient, limits the autograft approach. On the other hand, allografts can be rejected by the patient’s immune system [2,3]. Although these strategies have remained the gold standard for decades, regeneration encompassing full functional recovery of the target tissue or organ is hardly achieved.

To address these challenges, the field of TE and RM have emerged and benefited from important advances in multidisciplinary fields, including mechanical engineering, clinical medicine, genetics, materials science, engineering and life sciences [4]. The acceptance of tissue engineered constructs, such as three-dimensional (3D) scaffolds, has changed over the last few years. In the beginning, constructs were implanted and only then evaluated for their effects on tissue regeneration. As biotechnology has advanced, however, tissue specific requirements for implants were identified. Therefore, TE techniques for scaffold manufacturing had to become more refined so that the scaffolds could be tailored to each specific tissue’s needs (e.g., porosity, stiffness, resorbability, etc.) [4,5].

As was described by O’Brien et al. [4], to guarantee the suitability of a scaffold for TE applications, its biocompatibility, biodegradation, mechanical properties, architecture, and manufacturing technology must be considered. All of these factors are dependent on the selected biomaterial, which is the basis for scaffold fabrication. Over the years, different biomaterials have been explored for TE. Among these, synthetic polymers, such as poly(caprolactone) (PCL) and poly(lactic acid) (PLA), which present chemically defined compositions and exhibit tunable degradation rates, have been widely investigated [6,7]. While these are favorable properties for a biomaterial, synthetic polymers also present with limited mechanical properties, and due to their often hydrophilic nature, poor biocompatibility. As a result, there is a continuous effort to discover methods to improve upon these properties, and several options already exist; to name a few: by blending different polymer types, incorporating nanofillers or by modification through “click” chemistry. Out of these options, the incorporation of fillers is a common and effective procedure to improve a polymer’s physicochemical properties [8,9,10,11].

The ideal filler material already has outstanding properties on its own. An example of such a material is graphene (G), the elementary structure of graphite (Gt), appearing as a one-carbon-atom-thick sheet, and composed of sp^2^ carbon atoms arranged in a flat honeycomb structure. It is considered to be one of the lightest and strongest materials, with notable electrical and thermal properties, and hence, graphene-based materials (GBM) as a filler for several polymers has recently been explored. The different types of GBM can present different structures and properties and include graphene sheets (G), graphene oxide (GO), reduced graphene oxide (rGO), graphene quantum dots (GQD), and graphene nanoplatelets (GNP) [11,12]. Previous works have demonstrated that GBM are able to extensively improve the performance of several synthetic polymers on all fronts, even when incorporated in small quantities, while allowing the processing of such composite materials via advanced processing technologies [12,13].

These technologies range from electrospinning, which is a technique that uses a strong electrical field to obtain a scaffold of randomly oriented networks of polymeric nanofibers [14], to AM, which encompasses a group of technologies that create scaffolds in a layer-by-layer fashion with controlled architectures and properties [13,15]. The resolution is an important difference between these technologies, as electrospinning is characterized by lower fiber diameter than standard AM techniques. Ultimately, this has an effect on the porosity of the produced scaffolds, which influences stiffness, cell attachment, proliferation and differentiation [16,17,18]. Clearly, the fabrication method also plays an important role in defining the final properties, and thus the application of a scaffold. Due to the amount of variables involved, a large amount of literature has been produced on a wide variety of polymer/GBM composite scaffolds, presenting scaffolds of varied architectures, fiber diameters and compositions for use in different TE applications.

Here, we aim to comprehensively provide an overview of the current state-of-the-art in polymer/GBM composites for TE, while contrasting the fabrication techniques, to provide some perspective on the future directions of the field. First, an overview on synthetic polymers, GBM and their properties, as well as a summary of methodologies and fabrication techniques is provided, to appropriately focus the reader on the landscape of the field. Then, the fabrication of 3D polymer/graphene biocomposites is reviewed. The most recently available literature reports are included to highlight main applications in TE. Finally, the main conclusions and challenges of the fabrication of polymer/GBM biocomposites, together with the future perspectives, are presented.

## 2. Polymers

In the last few years, synthetic polymers have been vastly explored for TE applications. In contrast to natural polymers, synthetic ones possess a wider and more reproducible variety of physicochemical properties, such as tensile strength, elastic modulus, and degradation rate [19]. Due to their high tuneability, and high hydrolysability in the human body, aliphatic polyesters (e.g., PCL, PLA) embrace one of the most useful classes of synthetic polymers for TE [20,21]. This section briefly describes the synthesis, biodegradability, and biocompatibility of the most commonly used synthetic polymers in combination with GBM.

### 2.1. Poly(ε-Caprolactone)

PCL is compatible with a broad range of polymers and is approved by the Food and Drug Administration (FDA) for use in humans. Additionally, it has been widely used for AM due to its thermal stability, low melting temperature, and industrial-scale production, and is frequently combined with nanofillers for reinforcement [21,22,23].

#### 2.1.1. Synthesis

**Figure 1:**
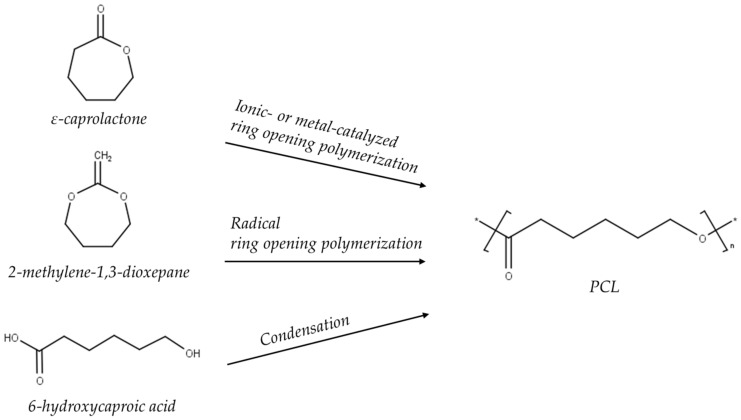
Different pathways for the synthesis of poly(ε-caprolactone) (PCL). Adapted from [27]. Copyright © 2022 Guarino et al. Published by the Encyclopedia of Polymer, Science and Technology.

PCL is synthesized by the polycondensation of 6-hydroxycaproic acid or ring-opening polymerization (ROP) of e-caprolactone (Figure 1). ROP is preferred, however, because the first method does not result in equally high quality yields. There are four types of reaction mechanisms (i.e., anionic, cationic, monomer-activated, coordination-insertion) through which ROP can occur, but this depends on the type of catalyst used in the reaction. Therefore several efficient catalysts (metal, organic, and enzymatic) have been used for that purpose. These affect PCL’s molecular mass and molecular mass distribution, end group composition, and copolymer’s chemical structure. The most-used catalysts during ROP of PCL are aluminum- or tin-based [22,24,25,26,27]. Overall, PCL is a versatile polymer since its chemical and mechanical properties can be further modified by copolymerization or blending [28,29]. PCL’s physicochemical properties are summarized in Table 1.

#### 2.1.2. Biodegradation

PCL biodegradation in the body is a bulk process that occurs in two stages. The first one concerns the hydrolytic cleavage of ester groups, which leads to a lower molecular weight (<3000). The second stage involves PCL intracellular degradation by phagosomes, giant cells or fibroblasts [22]. At high temperatures, PCL degrades by end-chain scission, while at low temperatures, it degrades by random chain scission [31]. It requires 2–4 years to be fully hydrolytically degraded [21,26].

#### 2.1.3. Biocompatibility

PCL biocompatibility has been evaluated over the short- and long-term. In general, no adverse reactions from the host tissue were reported [32,33]. For instance, Serrano et al. [34] studied the interaction of L929 mouse fibroblasts with a PCL film, and Corden et al. [35] examined the biocompatibility of PCL with osteoblast-like cells derived from human craniofacial bone; both groups found good adhesion, cell growth and viability in the presence of PCL. Lastly, aside from TE, it is worth mentioning that PCL is already used in a variety of (biocompatible) medical devices, such as sutures and wound dressings [22].

### 2.2. Poly(lactic acid)

PLA is an aliphatic polyester derived from renewable sources whose basic building block is lactic acid. It is highly versatile, biodegradable, biocompatible and it has extensive applications in the biomedical field, including TE [36]. Moreover, PLA and its copolymer PLGA have been approved by the FDA, which makes them very attractive for their use in biomedical products [37]. Despite the continuous development of PLA synthesis processes since 1932, it was only after the year 2000, with the beginning of PLA widespread commercialization, that its use in TE increased considerably [38,39].

#### 2.2.1. Synthesis

**Figure 2:**
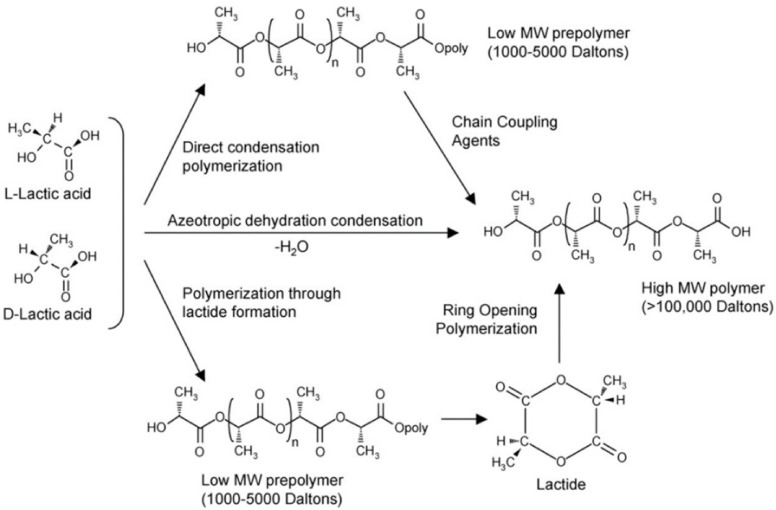
Mechanisms for poly(lactic acid) (PLA) synthesis. Reprinted from [43]. By Li et al. Published in *MDPI Molecules*.

Lactic acid (2-hydroxy propionic acid) is a chiral molecule, which exists in l- and d-enantiomers: poly-l-lactic acid (PLLA), poly-d-lactic acid (PDLA), and poly-d,l-lactic acid (PDLLA) [40]. Several reviews have addressed PLA synthesis [36,41,42,43], which involves lactic acid production, purification, and polymerization, as summarized in Figure 2. Direct polymerization and ROP are the most used. PLA synthesis demands precise temperature, pressure, and pH conditions, since its properties vary with isomer composition and reaction conditions [36]. Table 2 summarizes the polymer’s physicochemical properties.

#### 2.2.2. Biodegradation

PLA biodegradation involves hydrolysis of the ester linkage backbone, forming monomers or oligomers that are eliminated through the Krebs cycle [49,50]. Additionally, PLA degradation can be enzymatically enhanced in the presence of immune cells, which promote the biodegradation process by excreting acid phosphatase and lactate dehydrogenase [51]. Moreover, there is a discrepancy between the degradation rate of PLA’s enantiomers. For example, PLLA needs between 10 months and 4 years, depending on crystallinity, material geometry and molecular weight, to be completely degraded [52]. In general, PDLA degrades more rapidly than PLLA [53]. Therefore, the blending of enantiomers (l/d-PLA) is a method to tune PLA’s biodegradation.

#### 2.2.3. Biocompatibility

Many studies have been performed to evaluate its biocompatibility, especially in vitro. For example, Parks et al. [54] developed a 3D model consisting of human monocytes and fibroblasts to evaluate the inflammatory reaction of biomaterials such as PLA. They found significantly increased levels of inflammatory cytokines (IL-1β, IL-8, and TNF-α) after the introduction of PLA, which indicates an immune response in the cells as response to the material. This was not a bad result, however, as some inflammation is expected in the body’s natural response to facilitate wound healing. This is why in vivo implantation is vital to ultimately determine the safety of a material. Following this line of thought, Bos et al. [55] performed a study on the immune response of rats. The authors implanted PLLA samples subcutaneously in the backs of rats and observed them for a period of 143 weeks. After implantation, no chronic inflammatory reactions were reported and no implants were rejected. Lastly, it was estimated that the complete degradation of the samples would require more than 3 years in vivo.

## 3. Graphene-Based Materials

Since the Nobel Prize in Physics in 2010, graphene (G) has triggered tremendous attention within the scientific community. G is a two-dimensional (2D) crystalline material with sp^2^ hybridized atoms. It is the fundamental building block of hexagonally bonded carbon materials and consists of a 6-ring honeycomb lattice structure where each carbon atom is bonded to three neighboring atoms [56]. On a theoretical plane, if wrapped up, G forms a fullerene; if rolled up, it becomes a carbon nanotube (CNT); and when stacked (more than 10 G layers), it creates graphite (Gt) [57,58].

Other GBM described in the literature comprise few-layer G (2–5 G layers packed together) and multi-layer G (2–10 layers). The latter is also designated as G nanoplatelets (GNP). Each material can be submitted to several procedures, such as chemical oxidation, therefore creating G oxide (GO), few-layer G oxide, and GNP oxide (GNP-ox) [59].

**Figure 3:**
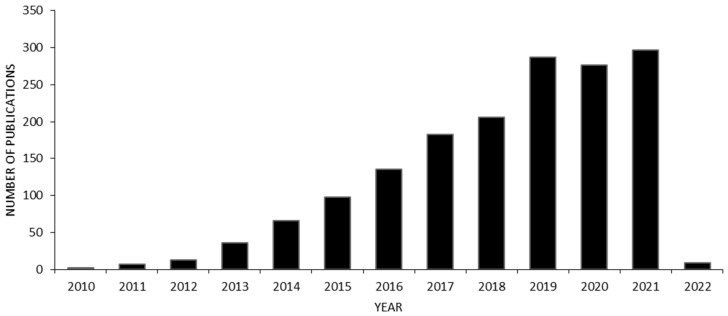
Number of publications concerning graphene in TE applications from 2010 to 2021; keywords: graphene, tissue engineering. [Source—Web of Science].

In addition to GBM’s wide variety, they present outstanding physicochemical properties and biological properties of interest [58]. Therefore, GBM are becoming a refreshing choice for biomedical applications, such as TE. Its trend of interest is outlined in Figure 3.

### 3.1. Production

In 1999, Ruoff et al. [59,60] exfoliated Gt into thin lamellae comprising multiple G layers via a micromechanical approach. In 2004, Geim and Novoselov [57,61] isolated G using a similar methodology. The novelty was based on the use of scotch tape to peel flakes from Gt. Since then, several studies have been focused on GBM production, optimization, and scale-up.

**Figure 4:**
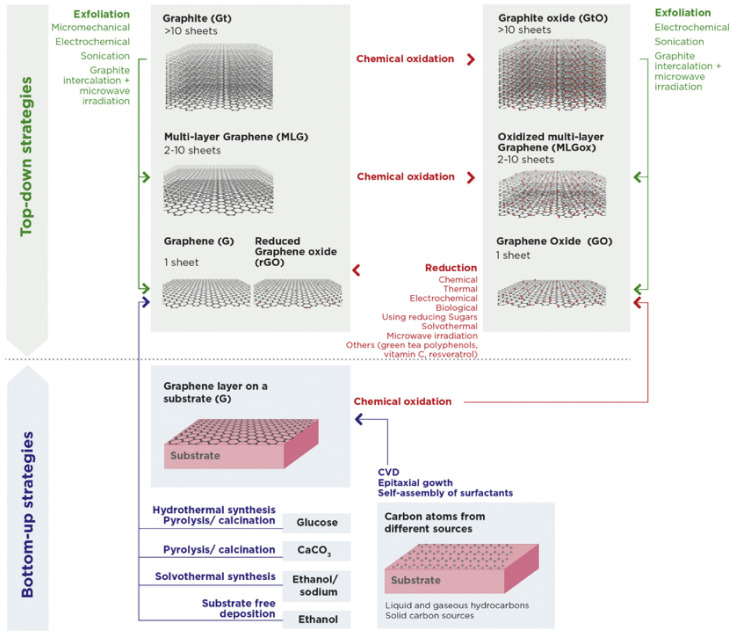
GBM family and its production methods. Reprinted from [11]. Copyright © Henriques et al., 2020. Published by Elsevier B.V.

G can be produced from top-down approaches using Gt as raw material, or bottom-up approaches using alternative carbon sources as a raw material. The top-down approaches involve exfoliation by intercalation, microwave irradiation or by electrochemical, micromechanical or sonochemical methods [11,60]. The bottom-up approaches create G either as a dispersion or powder (e.g., by chemical vapor deposition from liquid and gaseous hydrocarbons) or as a G layer on a substrate (e.g., by the reduction of glucose) [62]. The selected production methods all affect the final properties of the GBMs (i.e., size, thickness, functional groups) but the top-down methods are generally preferred because of simplicity and higher yields [11]. Figure 4 assembles the GBM types mentioned in the text, together with their corresponding production methods.

GBM oxidation disrupts aromatic ring hybridization, introducing oxygen in the form of hydroxyl and ether groups at the bulk surface, and carboxyl and carbonyl at the edges of the sheets, leading to an increase in hydrophilicity but also a decrease in electrical conductivity. GO is most commonly produced by the modified Hummers method, which, briefly, consists of stirring Gt powder with strong oxidizing agents, followed by sonication-mediated exfoliation [62].

The loss in electrical conductivity due to GBM oxidation can be partially recovered by reduction, yielding rGO, for example. There are several approaches to facilitate GBM reduction, including microwave irradiation and biological, (electro-)chemical or solvothermal methods. The use of green tea polyphenols, vitamin C, and resveratrol as biological methods are the most sustainable options. Nevertheless, the most-used are chemical and thermal approaches [63,64].

### 3.2. Physicochemical Properties

G stands out as a reference material since it owns plenty of unique properties, such as:

√ Being a one-carbon-atom-thick sheet (0.345 Nm) [65].
√ Having a remarkably low density of 0.77 mg/m^2^ [66].
√ Having an outstanding tensile strength of 130 GPa [67] and a Young’s modulus of ±1 TPa [68].
√ Possessing remarkable thermal conductivity (±4000 W/mK) [69].
√ Presenting very high electrical conductivity (10^4^–10^5^ S/m) [70].

Due to its unique properties, G has been shown to improve these specific properties of composite materials, even when incorporated in very low amounts, which establishes it as a promising material for the development of new composites for biomedical applications.

### 3.3. Biodegradation

As mentioned above, GBM oxidation usually involves the use of strong oxidants and concentrated acids. Despite this, there are several reports about graphitized materials being discharged into the environment [71]. Thus, novel eco-friendly approaches to promote GBM oxidation and degradation are being demanded.

GBM were considered structurally persistent until in vitro and in vivo studies provided evidence for its enzymatic degradation [72,73,74]. In detail, Kurapati et al. [72] demonstrated the biodegradation of single- and few-layer graphene by the human neutrophil-derived enzyme myeloperoxidase (hMPO) in the presence of low hydrogen peroxide (H_2_O_2_) concentrations. Further studies by the same authors showed that hMPO could also enzymatically degrade sheets of GO. In this work, the authors also observed a relation between GO biodegradability and colloidal stability (i.e., higher aggregation complicates GO degradation) [73]. Additionally, Mukherjee et al. [74] discovered that not only could the addition of purified hMPO degrade GO, but that the neutrophils themselves were also able to mediate the biodegradation of GO. This occurs through MPO-dependent extracellular degranulation, or in neutrophil extracellular traps (NETs). Moreover, the authors demonstrated the non-toxicity of degraded GO using a human bronchial epithelial cell line (BEAS-2B). Interestingly, however, attempts to degrade rGO with a similar horseradish MPO were unsuccessful [75].

### 3.4. Biocompatibility

Despite the recent work on the biodegradation of GBM, the mechanisms that lead to complete clearance from the body, as well as their biocompatibility, remain incompletely understood. Furthermore, this should be evaluated for each type of GBM separately, as they differ in chemical composition (e.g., degree of oxidation) and physical characteristics (e.g., dimensions, number of layers) [76].

In 2013, Pinto et al. [77] reviewed GBM biocompatibility and found reports on slight decreases in bacterial and mammalian cell viability after GBM exposure. Notwithstanding, the authors emphasized the need for further work in GBM long-term toxicity. More recently, in 2017, the same authors presented a preliminary assessment on PLA/graphene nanoplatelets (GNP) and PLA/functionalized carbon nanotubes (PLA/CNT-COOH) for anterior cruciate ligament reinforcement. Both in vitro and in vivo tests were performed. In the former, human dermal fibroblasts were seeded onto all formulations, and none exhibited cytotoxic responses. Moreover, each formulation supported cell proliferation for up to 3 days in culture. In the latter, nanocomposites were subcutaneously implanted in mice, and no severe inflammatory response was observed after 2 weeks of implantation [52]. In 2018, Fadeel et al. [78] reviewed the human and environmental safety assessment of GBM, mostly comprised of founding reports on GBM having minimal to no cytotoxicity. These authors presented the first steps toward a systematic collection of GBM biocompatibility data.

On a more systemic level, Jasim et al. [79] investigated the effects of thin, well-dispersed GO sheets on kidney function in mice after intravenous injection. Complete clearance from the body without nephrotoxicity was reported up to 1 month after exposure. Furthermore, in vitro experiments also confirmed the complete recovery of barrier function after 48-h GO exposure in endothelial cells and podocytes.

From a different perspective, Busy et al. [80] defined simple guidelines to ensure the safe usage of GBM in biomedical applications: reduce GBM dimensions (i.e., CNT < 5–10 μm length and 20 nm diameter, the use of G nanosheets) and assure good dispersion by realizing adequate surface hydrophilicity (e.g., hydrophilic surface functionalization for CNT and a high degree of oxidation for G).

Then, Bullock et al. [76] also stressed the relevance of avoiding GBM chemical contamination before biocompatibility evaluation, as any toxic compound used during GBM production, oxidation or reduction can remain bioavailable. For example, hydrazine, a reduction agent for GO, can have a cytotoxic and carcinogenic effect if not completely eliminated via a cleaning/purification process, although it can be replaced by more biocompatible compounds, such as l-ascorbic acid (vitamin C). Clearly, further identification of and substitution with greener alternatives is required.

Overall, GBM biocompatibility is a very broad topic and while some issues have been clarified, further studies are definitely necessary.

## 4. Polymer/GBM Composites

Polymer/GBM composites can be produced through numerous methods, most commonly via solution mixing, melt blending, in situ polymerization, and covalent bonding. Solution mixing consists of dispersing the GBM particles in a polymer solution, followed by solvent removal through evaporation to obtain a composite polymer film. Melt blending involves mixing a polymer melt and GBM powder under high shear conditions. In situ polymerization comprises the mixing of GBM in a solution of monomer and catalysts under the proper reaction conditions, so as to induce monomer polymerization. This allows for the possibility of covalent bonding between the polymer chains and the GBM surface [81,82].

The most-used methodologies are solution mixing and melt blending. The first may allow better GBM dispersion when affinity with the solvent is appropriate, which results in a good homogeneous composite, but is less environmentally friendly. The second allows large-scale and economical production of composites. However, it can result in less effective GBM dispersion in the polymer matrix, especially with high filler loadings. Therefore, some challenges still need to be addressed with this second type of polymer/GBM composite production, namely achieving homogeneous particle dispersion with minimal restacking, while optimizing interactions with the polymer matrix [83].

Nevertheless, these methodologies have been employed to produce highly performing composite materials. For example, Sayyar et al. [81] reported that incorporating chemically modified rGO through solvent mixing in PCL resulted in a doubled Young’s modulus and tensile strength, as well as a 14-fold increase in electrical conductance. Similarly, by solution mixing 0.5 wt.% modified G with PCL, Wang et al. [83] were able to improve the PCL’s Young’s modulus and yield strength both by approximately 12%.

Moreover, Gonçalves et al. [84] utilized melt blending to prepare PLA/GNP composites at several GNP loadings (0.1–0.5 wt.%). They obtained maximum mechanical performance with 0.25 wt.% GNP at specific mixing conditions (20 min, 50 rpm, 180 °C), for which PLA tensile strength, Young’s modulus, and toughness increased 20, 12, and 16%, respectively. For the higher loadings, the performance decreased due to GNP agglomeration creating defects within the polymer matrix.

In situ polymerization has also been extensively explored to prepare polymer/GBM composites. To illustrate, Yu et al. [85] prepared PCL/GO composites via in situ polymerization, and studied their effect as a nucleation agent, and indicated a 1.2-fold increase in crystallization temperature, adding 1.5 wt.% GO. Furthermore, Wang et al. [86] adopted this method to develop PCL/GO (0.5 wt.%) nanocomposites and observed 1.4-, 2.5- and 1.5-fold improvements in crystallization rate, tensile strength and Young’s modulus, respectively.

Clearly, graphene can enhance the key properties of several synthetic polymers when combined into new composite materials via the above-mentioned methods. While this creates an opportunity to explore new composites with improved properties, it also encourages their fabrication by a wide variety of techniques. These can be conventional techniques, (e.g., injection molding, solvent casting, particulate leaching, gas foaming, emulsion freeze-drying, thermally induced phase separation, electrospinning), but might also include additive manufacturing (AM) technologies (e.g., fused-deposition modelling (FDM), selective laser sintering (SLS), pressure-assisted microsyringe deposition (PAM)).

In general, conventional techniques are more user-friendly, cheaper, and as a result, more widespread. Unfortunately, simple techniques such as injection molding and solvent casting allow for very limited control over the complexity and architecture of the final structures. Moreover, it can be difficult to create identical samples with techniques such as injection molding, which depend on the quality of the used mold and skill of the user. Lastly, these methods can affect the homogeneity of used fillers in the produced samples. In contrast, AM techniques are more expensive as they require more sophisticated systems. However, they all facilitate the controlled fabrication of complex architectures of the final structures. Additionally, AM technologies can require high processing temperatures (e.g., FDM, SLS) that are unsuitable for some TE applications, such as biofabrication. Both conventional and additive fabrication methods have been extensively described in several reviews [14,15,20,87,88,89].

Despite this, electrospinning and AM are the most commonly described methods, and their mechanism will be elaborated on more in their respective subsections below, as we intend to discuss polymer/GBM composites fabricated by these two methods to illustrate their potential in TE applications.

### 4.1. Electrospinning of Polymer/GBM Composites

Electrospinning uses an electrical field to obtain polymeric fibers with a nanometric size diameter. Depending on the setup, it can process a wide range of materials from volatile solutions. The solution is charged by a high voltage while flowing through a needle and forming a polymer solution droplet (also known as Taylor cone). A polymer jet is formed when the potential difference surpasses the surface tension of the polymer solution. As a result, the long jet travelling distances results in whipping instabilities and the consequent deposition of random fiber networks. Due to the nanometric fiber sizes and dense networks (that approximate to the ECM of native tissues), electrospun meshes have been used for different TE applications. For example, anisotropically oriented electrospun nanofibers with the ability to mimic tendon behavior were already achieved [23,90], which builds towards the ultimate goal of promoting similar cell migration, proliferation, and differentiation as in native tissues. This, combined with its cost-effectiveness, simplicity, and versatility, are some of the reasons for the increasing popularity of electrospinning in TE [87].

Thus, it is not surprising that electrospinning has also been used extensively for the fabrication of polymer/GBM composite scaffolds. However, this can come at the cost of printability since the addition of a GBM phase will also affect the viscosity and conductivity of the solution. In general, a higher viscosity will result in thicker fibers, whereas increases in conductivity lead to more stretching of the fibers if the printing parameters are not adjusted accordingly [91]. Nevertheless, the ability to create polymer/GBM scaffolds with submicron fiber diameter by electrospinning makes it possible to cater to a wider set of requirements (i.e., architecture by electrospinning and material properties of polymer/GBM composites) of tissues.

**Figure 5:**
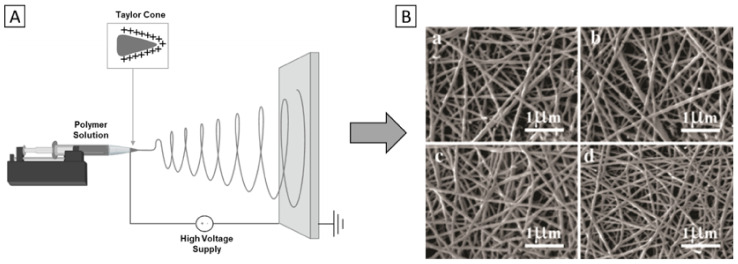
Electrospinning of bone TE scaffolds. (**A**) Schematic representation of the electrospinning process. (**B**) Scanning electron microscopy (SEM) images of PCL/chitosan/collagen/GO composite scaffolds; (**a**) 0 wt.% GO, (**b**) 0.5 wt.% GO, (**c**) 3 wt.% GO, (**d**) 6 wt.% GO. Reprinted (**B**) from [92]. Copyright © Aidun et al., 2019. Published by the International Center for Artificial Organs and Transplantation and Wiley Periodicals, Inc., Painesville, OH, USA.

For bone TE in particular, numerous studies have already been conducted. For example, Aidun et al. [92] recently manufactured PCL/chitosan/collagen/GO biocomposites and reported enhanced osteogenic properties. By solvent mixing, GO was added to PCL/chitosan/collagen to obtain solutions with concentrations of 0.5, 3, and 6 wt.%. Subsequently, electrospinning was performed using a set-up schematically represented in Figure 5A.

Interestingly, a decrease in mean fiber diameter and pore size with increasing GO percentage was observed for the obtained scaffolds (0 wt.% GO: 128 nm; 6 wt.% GO: 115 nm), as seen in Figure 5B. This was attributed to increased viscosity and conductivity of the electrospinning precursor solution as a result of its GO content. Furthermore, with respect to material properties, the hydrophilicity and swelling capacity of the composite was most improved in the 6 wt.% GO group. Similarly, after a 28-day incubation period in simulated body fluid, the 6 wt.% GO scaffolds facilitated the most hydroxyapatite (HA) sedimentation. Lastly, energy-dispersive x-ray (EDX) spectra measured a Ca/P ratio of 1.68, which approximates human bone values.

**Figure 6:**
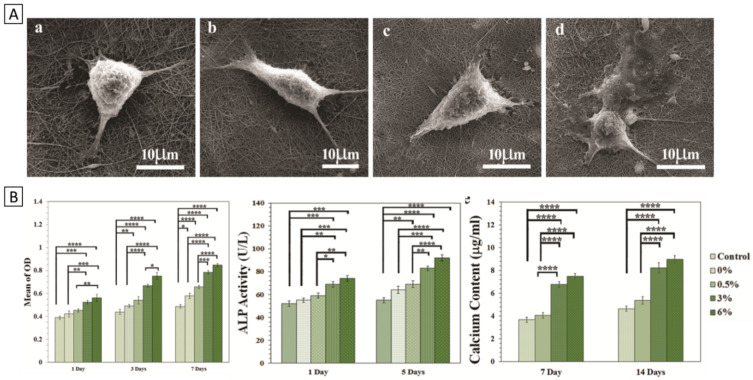
Biological evaluation of PCL/chitosan/collagen/GO scaffolds over a period of 14 days shows improved osteogenic capacity of GBM composite scaffolds. (**A**) Cell attachment onto scaffolds of (**a**) 0 wt.% GO, (**b**) 0.5 wt.% GO, (**c**) 3 wt.% GO, (**d**) 6 wt.% GO. (**B**) Left-to-right: quantified cell viability, ALP expression and calcium deposition on the scaffolds. (* *p* < 0.05, ** *p* < 0.01, *** *p* < 0.001, **** *p* < 0.0001). Reprinted from [92]. Copyright © Aidun et al., 2019. Published by the International Center for Artificial Organs and Transplantation and Wiley Periodicals, Inc., Painesville, OH, USA.

Then, in vitro experiments were performed with the human osteosarcoma (MG-63) cell line, which are displayed in Figure 6. Overall, better cell attachment and proliferation were observed with increasing amounts of GO, which is in accordance with the higher hydrophilicity of the PCL/chitosan/collagen/GO (6 wt.%) scaffolds. The authors state that this was because of increased protein adsorption affinity derived from GO oxygen-containing functional groups. The PCL/chitosan/collagen/GO scaffolds were then evaluated for their osteogenic capacity by performing an alkaline phosphatase (ALP) assay and measuring calcium deposition, as early and late osteogenic markers. In line with previous findings, it was found that at higher amounts of GO, increased ALP activity and calcium deposition were observed. However, there was no significant difference between 3 and 6 wt.% of GO. Therefore, the authors concluded that PCL/chitosan/collagen scaffolds should be combined with at least 3 wt.% GO to effectively improve these composite scaffolds’ osteogenic capacity.

Similarly, Marrella et al. [93] developed and compared PCL/GO and PCL/rGO scaffolds for bone TE applications. The authors evaluated the influence of GO and rGO on the mechanical, physicochemical and biological properties. By introducing GO and rGO (0.25 wt.%), the Young’s modulus of PCL increased by 23% and 38%, while tensile strength increased by 48% and 16%, respectively. Biological assays were performed with fibroblasts and osteoblast-like cell lines. For rGO, increased bone cell viability and proliferation was reported, as well as improved cell spreading, likely because rGO facilitates better mineralization than GO due to its increased surface roughness.

Altogether, in both instances, the incorporation of GBM has favorably improved the properties of composite scaffolds for bone regeneration, which illustrates the high potential of GBM in bone TE applications.

GBM and their composites also find applications in cardiac tissue engineering. Currently, the major worldwide cause of death is cardiovascular disease [94]. As a result of the limited regeneration capacity of cardiac tissue, any injury or damage to it may become permanent. Therefore, the development of biocomposites to regenerate heart valves, vascular grafts, and heart stent components is needed.

In this regard, Hitscherich et al. [95] explored the potential of 3D nanofibrous PCL/G scaffolds for cardiac TE. By sonication, G-nanoparticles (≈70 nm) were dispersed within PCL solutions at concentrations between 0.005 and 0.5 wt.% of G. Subsequently, nanocomposite scaffolds were manufactured with randomly oriented fibers at average fiber diameters of ≈430 nm and ≈630 nm for 0.005 and 0.5 wt.% of G, respectively. The even distribution of G-nanoparticles over the fibers was confirmed when the authors measured a decrease in impedance at higher G concentrations. Moreover, the authors reported that a higher amount of graphene resulted in higher biocomposite conductivity, namely ~1 × 10^−13^ S/cm for PCL, ~1.5 × 10^−13^ S/cm for PCL/G (0.01 wt.%) and ~1.5 × 10^−10^ S/cm for PCL/G (0.05 wt.%). Most likely, this improvement was achieved because the electroactive G-nanoparticles provided local conductive sites in the polymer matrix.

Biological assays were also performed by seeding mouse embryonic stem-cell-derived cardiomyocytes (mES-CM) onto these PCL and PCL/G scaffolds. In all groups, mES-CMs adhered well, spread out within the first 24 h of in vitro culture and displayed well-defined sarcomeres. Spontaneous beating of the mES-CMs was observed after 48 h, achieving synchronous contraction across the scaffold after approximately 4–5 days. However, cells on PCL/G scaffolds exhibited significantly lower spontaneous beating frequency; a sign of further differentiation. Furthermore, in G-enriched groups, caffeine-transient T_50_ was significantly decreased, possibly indicating the involvement of Ca^2+^ efflux mechanisms. Therefore, the authors suggest that graphene plays a role in the expression or organization, or both, of the sarcolemmal Na^+^/Ca^2+^ exchanger (NCX), which can facilitate faster Ca^2+^ homeostasis via expulsion from the cytoplasm. This would also provide an explanation for the reduced spontaneous beating frequency of PCL/G scaffolds. Additionally, the authors worked with a different rotating collector set-up described elsewhere [96]. This method enables the production of scaffolds with axially oriented fiber morphology. The biggest improvements in cardiac specific protein and contractile behavior were reported in aligned PCL/G scaffolds, such as the upregulation of Cx43, suggesting enhanced cell–cell coupling and the highest fractional release, a measure for excitation–contraction coupling efficiency. This emphasizes the versatility of electrospinning in generating nanofibrous scaffolds, but also the need for controlled fiber orientation for improved biological outcomes.

For eventual use in treatment of atrioventricular blocks, Chen et al. [97] evaluated gelatin/PCL/G scaffolds both in vitro and in vivo. Briefly, a biocomposite scaffold was developed via electrospinning with increasing G-nanoparticle contents, up to 1 wt.%. The incorporation of G improved scaffolds’ mechanical and electrical properties, but enlarged the fiber diameter; to illustrate: Young’s modulus increased from 28.74 ± 3.35 MPa (0 wt.%) to 37.20 ± 6.37 MPa (1 wt.%), conductivity increased from 0.15 × 10^−3^ S/cm (0 wt.%) to ~11.15 S/cm (1 wt.%) and fiber diameter was enlarged from 489 ± 68 nm (0 wt.%) to 595 ± 119 nm (1 wt.%).

Then, the authors assessed scaffolds’ in vitro biocompatibility. Neonatal rat ventricular cardiomyocytes (NRVCMs) were isolated and seeded onto the developed scaffolds. It was demonstrated that after 5 days, cells adhered and spread on the scaffolds up to 0.5 wt.% of G. However, some cytotoxicity was reported at higher G concentrations. For this reason, further in vivo studies were performed by implanting the intermediate concentration PCL/gelatin/G (0.5 wt.%) scaffolds into rats for a period of 12 weeks and evaluating for toxicology. No signs of chronic inflammation or other detectable adverse reactions were reported after 4, 8 or 12 weeks. Both authors show that the usage of G-nanoparticles can significantly, and safely, improve the potential of electrospun biocomposites for cardiac TE.

Artificial nerve guidance conduits (NGC) have also been developed to mimic the nerve’s natural structures and components. However, as the efficacy of NGCs is limited, more research on neural TE is needed. In this regard, Fang et al. [98] developed a PCL/Gelatin methacryloyl (GelMA)/rGO NGC with a range of rGO concentrations (0, 0.25, 0.5, 0.75, 1.0 wt.%), and evaluated both their in vitro and in vivo biological effects. Fibers were obtained in a randomly oriented arrangement, forming a 3D interconnected porous network. Surprisingly, the addition of rGO to the PCL/GelMA composite did not increase fibre diameter, as an average fibre diameter of 400 nm was measured across all groups. By incorporating rGO into NGC, its electrical conductivity increased from ~2.0 mS/cm to ~9.3 mS/cm. Mechanical properties were not significantly improved by the addition of rGO.

Biological activity was firstly evaluated in vitro. The hybrid scaffolds with low concentrations of rGO (0.25 and 0.5 wt.%) significantly improved the proliferation of Schwann cells (RSC96), whereas again at higher concentrations, there was some cytotoxicity. The authors also reported a significant upregulation of Sox2, which is one of the Yamanaka factors [99] responsible for reprogramming fibroblasts to induced pluripotent stem cells (iPSCs), in cells cultured on the PCL/GelMA/rGO (0.5 wt.%) scaffolds compared to 0 wt.% control. As the possibility to reprogram Schwann cells to more stem-like cells was previously demonstrated by Masaki et al. [100], the authors carefully conclude that hybrid rGO scaffolds might similarly be able to induce a mesenchymal-like phenotype.

Then, PCL/GelMA/rGO (0 wt.%, 0.5 wt.%) and autograft nerves were implanted in a 10 mm rat sciatic nerve defect model and evaluated in vivo. After a period of 12 weeks, no signs of inflammation were found, and all wounds healed without complications. Recovery of the sciatic nerve was evaluated by walking track analysis, muscular atrophy and electrophysiology. On all levels, the 0.5 wt.% PCL/GelMA/rGO group performance almost rivalled the autograft group, which is regarded as the gold standard. The authors note, however, that through electrical stimulation, the peripheral nerve could be stimulated to regenerate even further.

This example of using GBM in neural TE can be the basis for further research on nerve regeneration, and shows GBM’s great potential in the field. More examples are included in the table below (Table 3), which summarizes several works on biocomposites fabricated via electrospinning. It contains examples with applicability from hard to soft TE that take advantage of G’s unique features, namely mechanical, thermal and electrical properties.

### 4.2. Processing of Polymer/GBM Composites by Additive Manufacturing

In contrast to electrospinning, AM technologies afford the development of patient-specific scaffolds with intricate configurations and specific properties for TE. The most commonly used AM techniques comprise melt extrusion-based methods, such as fused-deposition modeling (FDM), and solution/slurry extrusion-based methods, such as pressure-assisted microsyringe (PAM) deposition [122]. FDM employs one (or more) temperature controlled nozzles that melt different polymers; the heated nozzle is computer-controlled and typically moves in x- and y-direction to precisely deposit the molten filament into a predefined pattern. As the layer is completed, the nozzle moves up (z-direction) and begins the next layer. The main disadvantage of the technique is its reliance on the melting of thermoplastic polymers at temperatures (>37 °C) which prevent the incorporation of living cells or growth factors during printing [122]. Alternatively, if biofabrication is the goal, PAM and other extrusion-based systems are used, which can also process at lower temperatures by using assisted-extrusion, with the cost of reduced resolution out of the printing plane (z-direction). The use of such AM techniques for the fabrication of polymer/GBM composite scaffolds has resulted in well-defined architectures. However, while these printing methods are not affected by the change in conductivity caused by the incorporation of GBM, they inherently have a more limited resolution compared to electrospinning. Therefore, these additively manufactured polymer/GBM scaffolds are characterized by their strength and complex, controlled architectures, at the cost of some porosity.

**Figure 7:**
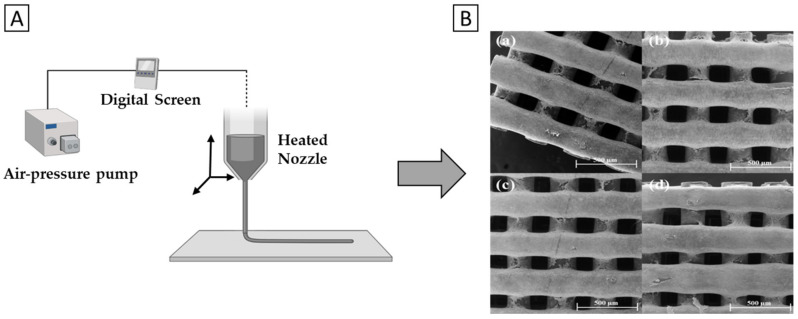
Additive manufacturing (AM) of (bone) TE scaffolds. (**A**) schematic representation of the AM process. (**B**) SEM images of PCL/G composite scaffolds at different concentrations; (**a**) 0% G, (**b**) 0.13% G, (**c**) 0.5% G, (**d**) 0.78% G. Reprinted (**B**) from [124]. Copyright © Wang et al., 2019. Published by Elsevier.

For example, Wang et al. [123] developed a nanocomposite scaffold of PLA/GNP/l-Arginine (l-Arg) with enhanced mechanical and biological properties by FDM. First, GNP was functionalized with l-Arg to improve its compatibility and dispersion throughout the PLA matrix, after which PLA and different amounts of GNP/l-Arg (0.5, 1, 2, 4, 6, 8, 10 wt.%) were mixed. Subsequently, the nanocomposite structures were 3D printed at an average fiber diameter of 400 µm (Figure 7). The 3D printed samples at different loadings of GNP/l-Arg displayed uniform diameters and strong adhesion between successive layers. The fiber cross-sections of empty PLA scaffolds were of smooth and brittle morphology, whereas PLA/GNP and PLA/GNP/l-Arg scaffolds were rougher. No agglomeration of GNP or GNP/l-Arg was found up to a content of 2 wt.%, while at higher GNP/L-Arg contents (>2 wt.%), tensile and flexural strength declined. As a result, optimal mechanical properties were observed at a GNP/L-Arg loading of 2 wt.%, specifically 67.2 MPa for tensile strength and 105.4 MPa for flexural strength, which was an increase of 43.6% and 28.5%, respectively, when compared to pristine PLA. Furthermore, PLA/GNP/l-Arg always displayed a higher strength than unmodified PLA/GNP.

Lastly, cytotoxicity assays using L929 cells cultured on GNP/l-Arg (0, 0.5, 1, 2, 4 wt.%) scaffolds also demonstrated the best cell viability in the 2 wt.% groups, with an 18% improvement in cell viability versus empty PLA scaffolds. Overall, these promising results demonstrate a potential for the use of additively manufactured polymer/GBM composite scaffolds in TE.

Moreover, Wang et al. [124] fabricated PCL/G scaffolds and thoroughly evaluated its biological properties in vitro and in vivo. G nanosheets were mixed into the PCL phase by melt blending into three final concentrations (0.13, 0.50 and 0.78 wt.%) and the constructs were subsequently fabricated with a commercial, screw-assisted AM system with a constant fiber diameter of 330 µm.

**Figure 8:**
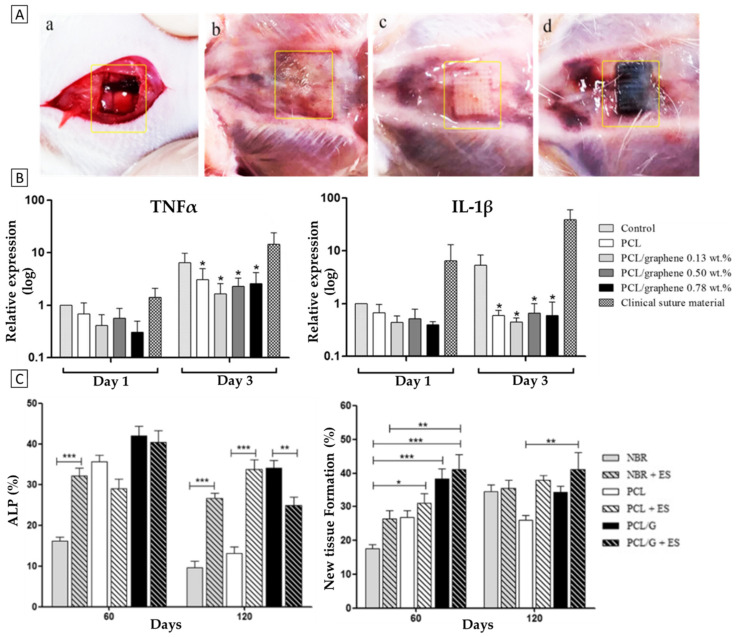
Biological evaluation of PCL/G (0, 0.13, 0.50, 0.78 wt.%) scaffolds over a period of 4 months in vivo. (**A**) Rat calvaria, critically-sized defect model; (**a**) during surgery, empty, (**b**) 120 days post-surgery, empty, (**c**) 120 days post-surgery, PCL scaffold, and (**d**) 120 days post-surgery, PCL/G scaffold. (**B**) Left-to-right: TNF-α expression as indicator of immune response, ALP expression as an early osteogenic marker, the quantification of new tissue formation (* *p* < 0.05; ** *p* < 0.01; *** *p* < 0.001). Adapted from [124]. © Wang et al., 2019. Published by Elsevier.

Before implantation, the scaffolds were assessed in vitro on cell viability assays with mouse pre-osteoblastic cells (MC3T3) and immune response tests with human monocytic cells (TIB-202). Regarding cell viability, it was found that increasing graphene concentrations correlated to increased cell viability and proliferation. Indeed, cell proliferation rates were significantly higher in 0.5 and 0.78 wt.% PCL/G than in PCL scaffolds (Figure 8B), while bridging of the cells between the pores was observed. Furthermore, to assess the immune response, scaffolds were compared with clinical suture materials as a control, by measuring the expression of inflammatory factors (TNF-α, IL-1) for 3 days. The authors observed significantly lower expression of TNF-α and IL-1 than in the control for all groups, which indicated no immune response to the introduction of the scaffolds, therefore suggesting a high potential for PCL/G scaffolds in in vivo applications.

Then, to follow up on their suggestion, the authors studied the osteogenic effects of the PCL/G scaffolds in a rat calvaria, critically-sized defect model (Figure 8A). Additionally, the effect of electrical stimulation (ES) was studied, since it has been previously demonstrated that electrical microcurrent stimulation (10–20 µA) can further promote osteogenesis [125]. For such applications, the conductivity of GBM is relevant. After 2 and 4 months post-implantation, histological assays were performed to examine new tissue formation. No signs of inflammation were observed in any group. Additionally, more organized bone formation, greater portions of new bone and further tissue maturation were observed in all PCL/G groups (Figure 8B). Lastly, the expression of four osteogenic factors (ALP, RANK, RANKL and OPG) was monitored, where all groups had increased expression compared to the control group (NBR), of which ALP is illustrated in Figure 8B. Notably, the authors ultimately conclude that ES, in combination with PCL/G scaffolds, is the most effective method of inducing new tissue formation. To summarize, this study has demonstrated the tremendous potential of GBM scaffolds to promote in vivo tissue formation, and has shown how the GBM’s electrical properties can be exploited in combination with other treatment methods, such as ES, to promote tissue repair.

Interestingly, Hou et al. [126] attempted to develop a dual-function biocomposite of PCL/G by screw-assisted extrusion printing for the treatment of bone cancer, as well as subsequent tissue regeneration post-treatment. The PCL/G biocomposite was achieved via melt blending by adding G at different concentrations (5, 7, 9 wt.%). Similar to observations from electrospinning, the incorporation of a higher G amount resulted in a higher fiber diameter. PCL scaffold mean fiber diameter was 336.4 ± 8.4 µm, while for PCL/G (9 wt.%) it was 363.2 ± 23.1 µm. Results from thermogravimetric analysis showed that no significant losses in G content occurred during melt blending or the printing process. Furthermore, through mechanical analysis, it was found that the presence of 6 wt.% of G considerably increased the compressive modulus and strength by 42% and 40%, respectively. These approximated the compressive modulus and strength of human trabecular bone (56 ± 29.6 MPa; 3.9 ± 2.7 MPa) [127].

However, biological studies performed were less successful. Human adipose-derived stem cells (hADSCs) and sarcoma osteogenic cells (SAOS-2) were seeded on the scaffolds to establish a relationship between G content and hADSCs/SAOS-2 survival rate. After 3 days of seeding, only SAOS-2 proliferation was significantly reduced, and this effect (while slighter) was also significantly present in the hADSCs after 7 days of in vitro culture. Therefore, the authors had to unfortunately conclude that achieving a dual-functional scaffold to both treat bone cancer and regenerate bone was too ambitious. However, they do state that the additively manufactured PCL/G scaffold has interesting mechanical properties for bone TE and that it does have potential as a bone cancer treatment option, separately. Other similar state-of-the-art studies of AM techniques for the development of polymer/GBM biocomposites and scaffolds are summarized below in Table 4.

## 5. Overview and Conclusions

**Figure 9:**
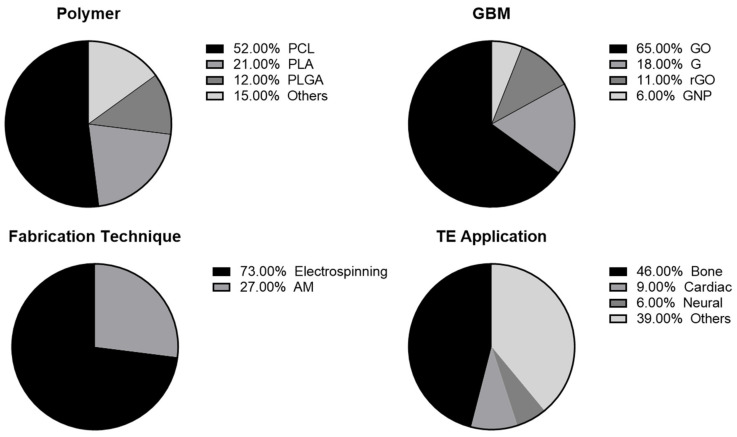
Overview on the most used polymers, graphene-based materials (GBM), fabrication techniques, and tissue engineering (TE) applications reported.

By presenting the most recently available literature about polymer/graphene biocomposites produced by advanced processing technologies in TE, this review has highlighted graphene’s ability to improve synthetic polymer’s biological, electrical, mechanical (namely Young’s modulus and tensile strength), and thermal properties. Furthermore, it depicts representative examples of polymer/graphene biocomposite applications for several tissue types. Figure 9 presents an overview on the most-used materials, techniques and TE applications reported.

PCL was the most commonly used polymer, comprising 52% of the polymer-GBM composites. This is a considerable difference as PLA, the next most-used, accounted for 21%. While PLA does exhibit slightly better mechanical properties, the reason for PCL being more commonly used might be the fact that it possesses a lower melting point, which facilitates processing. It could also be explained by the stand-alone FDA approval of PCL, as PLA has so far only been approved in combination with other products. Moreover, this may simply be the case because of the later commercialization and widespread use of PLA. Nevertheless, both polymers exhibit very similar properties, and they can be used either separately or in combination, depending on the desired outcome.

Out of all the types of GBM, GO was the most used (65%), being incorporated in polymers in amounts of approximately 0.3–2.3 wt.%. The oxidation of G makes this particular GBM more hydrophilic, therefore resulting in increased biocompatibility, which is clearly regarded as a very important property for TE. The electrical properties of GBM are important for specific applications (e.g., neural, cardiac, bone), which is where the reduction of GO is of interest. In this regard, some of the presented studies in this review did report that rGO more successfully supported cell viability, cell spreading and proliferation for neural and osteoblast lineages. Thus, since GBMs have a versatile range of properties, the most relevant type of GBM should be selected for the intended TE application.

Overall, the final application determines the specific scaffolds’ requirements, and this holds true for composites of any combination of materials, of which GBM composites are no exception. Therefore, composites in general have the aim of improving properties to better meet tissue-specific requirements. Improved tensile and compressive strength, cell proliferation and differentiation into a desired lineage were the most reported effects after GBM incorporation. Moreover, bone was the most studied tissue application for polymer/GBM composite scaffolds (49%), along with cardiac (9%) and neural (6%) tissues; the ‘others’ group mostly consisted of scaffolds developed without a specific application in mind. It is the electrical properties of GBM that distinguishes it as an attractive option for bone, cardiac and neural TE applications. However, to accurately evaluate the effectiveness of polymer-GBM composite scaffolds for biomedical applications, there is still the need for more in vivo studies to be performed in the field. Some promising results were discussed in this review, yet overall there is only limited data available. This is essential to help take the next major step towards advancing the TE field and to ultimately translate polymer/GBM composites to use in clinics.

Eventually, with the development of novel advanced manufacturing techniques, it is expected that the manufacturing of polymer/GBM composite scaffolds will also considerably profit. Currently, electrospinning has been by far the most described fabrication technique for polymer/GBM composite scaffolds (73%), likely because it allows one to obtain nanoscale fibers in randomly or anisotropically arranged networks. This is favorable because it results in more porous scaffolds, which are shown to promote cell attachment, cell infiltration, diffusion and degradation. However, the precise deposition of fibers will be of equal importance in manufacturing functional tissues, specifically tissues that derive their function from their complex architecture (e.g., kidneys, heart). Therefore, ideally, it would be possible to print in a precisely controlled manner, at a high resolution. It was for this reason that the development of melt-electrowriting (MEW) technology, which is both capable of accurate fiber deposition and achieving submicron fiber diameter, was very well received. Another prospect would be the integration of different techniques into one platform, where more manufacturing technologies are combined to fabricate more functional tissue structures. This provides a relevant topic for further research into functional, high resolution, polymer-GBM composite scaffolds and for the advent of further applications.

## Figures and Tables

**Table 1:** Summary of PCL physicochemical properties.

Property	Unit	Range	Reference
Crystallinity	%	<69	[28]
Density	g/cm^3^	1.07 to 1.20	[28]
Decomposition temperature	°C	300 to 350	[28]
Glass transition temperature	°C	−65 to −61	[22,28]
Melting temperature	°C	56 to 65	[8,22,30]
Elongation at break	%	20 to 1000	[30]
Tensile strength	MPa	20.7 to 42	[30]
Young’s modulus	GPa	0.21 to 0.44	[21,22]

**Table 2:** Summary of PLA physicochemical properties.

Property	Unit	Range	Reference
Crystallinity	%	<35	[44]
Density	g/cm^3^	1.21 to 1.25	[45]
Decomposition temperature	°C	300 to 370	[30]
Glass transition temperature	°C	50 to 65	[45,46,47]
Melting temperature	°C	150 to 178	[30,46,47]
Elongation at break	%	2 to 160	[30,46,47]
Tensile strength	MPa	6.6 to 60	[46,47,48]
Young’s modulus	GPa	0.35 to 3.5	[46,47,49]

**Table 3:** Biocomposites fabricated via electrospinning, along with the printing condition (flow rate, distance to collector (D_t_C), applied voltage (V), and fiber diameter (F_d_)) applications, and outcomes.

Polymer	Filler (wt.%)	Other Elements	Flow Rate (mL/h)	D_t_C (cm)	V (kV)	F_d_ (nm)	Application	Outcomes	Ref.
PCL	GO (3, 6)	Chitosan/ Collagen	0.6	12	20	120	Bone TE	↑ Hydrophilicity (WCA ↓ to 52°) ↑ GO amount = ↑ MG-63 cells’ attachment and proliferation	[92]
PCL	GO rGO (0.25)	-	2.0	12	10	430 410	Bone TE	Young’s modulus: ↑ 23% (GO) and 38% (rGO) Tensile strength: ↑ 48% (GO) and 16% (rGO) rGO was more efficient, ↑ cell viability and proliferation	[93]
PCL	G (0.01, 0.5)	-	1.5	15	17	<1 × 10^3^	Cardiac TE	Volume conductivity ↑ from 1 × 10^−13^ to 1.5 × 10^−10^ S/cm ↑ Cardiomyocytes spontaneous contraction	[95]
PCL	G (<0.5)	Gelatin	2.0	12	15	600	Cardiac TE	In vitro: ↑ Neonatal rat ventricular myocyte growth and survival rate In vivo: After implanting into rats for up to 12 weeks, inflammation was not assessed	[101]
PCL	rGO (<1)	GelMA ^1^	2.0	15	15	400	Neural TE	↑ Schwann cell proliferation ↑ Nerve regeneration and functional recovery	[98]
PCL	GO (0.1, 1)	-	1.0	12	18	400	Control cell behavior	~20% ↑ in tensile strength (up to 0.3 wt.% of GO) ↑ Adhesion, spreading, and differentiation of mouse mesenchymal stem cells (mMSCs) into osteoblast-like cells	[102]
PCL	GO (0.5)	M_g_O	1.0	10	18	700	Bone TE	↑ Adipose-derived stem cell adhesion and viability	[103]
PCL	GO (<0.4)	Gelatin	-	-	-	135	Neural TE	Antibacterial potential: No bacterial (*Escherichia (E.) coli* and *Staphylococcus (S.) aureus*) growth was observed Suitable microenvironment for rat cell migration, adhesion, and proliferation	[104]
PCL	GO (0.5)	Quercetin	0.5	15	18	300, 500	Wound healing	Quercetin maximum release ↑ to 70% after 15 days ~50% ↓ in bacterial growth after 12 h Fibroblast cell viability was 95%	[105]
PCL	GO (0.1)	Dexamethasone	0.8	10	18	166	Bone TE	2-fold ↑ in osteogenic differentiation ability	[106]
PCL	GO	-	0.5	15	20	100	Skeletal muscle TE	~30% ↓ in skeletal muscle cell elongation ability	[107]
PCL	GO (0.5, 4)	PU ^2^	0.3	15	9, 10	400, 600	Skin TE	↑ Hydrophilicity (WCA ↓ to 80° Cytotoxicity was not characterized	[108]
PLA	GO (10)	Ionic liquid	0.5	20	15	<1.8 × 10^3^	Tracheal TE	Antimicrobial potential: Scaffolds’ IC_50_ against *E. coli* and *S. aureus* ↓ from 55 and 48 μg/mL to 0.8 and 0.76 μg/m ↑ Fibroblast attachment, infiltration, and proliferation In vivo: successful implantation into rabbit models	[109]
PLLA	GO (1)	BMP2 ^3^	1.0	20	20	700	Bone TE	↑ Protein adsorption ↑ Adipose-derived stem cell attachment and proliferation ↑ Expression of bone-related markers	[110]
PLGA ^4^	GO (2)	Poly-L-Lysine	(4.2, 6.0)	20	40	<1.5 × 10^3^	Bone TE	↑ Hydrophilicity (WCA ↓ by 13%) ~118% ↑ in tensile strength Electric stimulation (0.5 V) enhanced osteogenic differentiation	[111]
PLGA	GO (2)	HA	1.0	20	20	<1 × 10^3^	Bone TE	~2-fold ↑ in tensile strength ↑ Osteoblastic cell (MC3T3-E1) adhesion and proliferation ↑ Expression of bone-related markers	[112]
PLGA	GO (2)	Gelatin	1.0	20	20	<1 × 10^3^	Bone TE	↑ MC3T3-E1 adhesion and proliferation ↑ Expression of bone-related markers	[113]
PLGA	GO	RGD peptide	0.2	11	14	558	Smooth muscle TE	↑ Hydrophilicity (WCA ↓ to 80°) Thermal stability was not affected ↑ Vascular smooth muscle cell attachment and proliferation	[114]
PLGA	GO	IGF-1 + BDNF ^5^	(4.2, 6)	10	40	1 × 10^3^	Spinal cord injury	In vitro: ↑ Neural stem cell proliferation and differentiation Oxidative stress was not verified In vivo: ↑ Functional locomotor recovery ↑ Number of neurons at the injury site	[115]
PLGA	GO (1)	-	-	20	10	<1.5 × 10^3^	Tendon to Bone Integration	13% ↓ in tensile strength In vitro: ↑ in rabbit bone MSCs (after 3 days), ALP activity (days 7 and 14), and osteogenic ability (after 14 days) In vivo: ↑ the ability to form new bone at the tendon–bone interface and promote supraspinatus tendon-to-bone integration (bone mineral density ↑ ~12% at 12th week)	[116]
PU	GO (0.5, 1)	PEG ^6^	0.4	11	18	(322, 1 × 10^3^)	Skin TE	~52% ↑ in ultimate strength ~6% ↑ in tensile strength After implanting into (Albino Wistar) rats for up to 3 months, inflammation was not studied	[117]
PU	GO (<8)	Polycarbonate diol	2.0	10	12, 5	<1 × 10^3^	Skeletal muscle TE	↑ Hydrophilicity (WCA decreased by 50% after 10 min) Upregulation of myogenic mRNA levels ↑ Expression of myosin heavy chain	[118]
PVA ^7^	G (<3)	-	0.2	-	15, 19	<100	Cardiac TE	↑ Endothelial cell adhesion and proliferation (over 4 days) Cytotoxicity was not characterized	[119]
PVA	rGO (0.1, 1)	Glucose + Glutaraldehyde	1.6 × 10^−4^	15	(16, 18)	200	Skin TE	↑ Metabolic activity after cell culture for 21 days Cytotoxicity was not verified	[120]
PVP ^8^	GO (<2)	Chitosan + Polyethylene	-	-	(20, 24)	60	Wound closure	In vitro: ↑ mMSC attachment and viability up to 72 h In vivo: (adult male Sprague Dawley rats) faster wound closure rate (about 33%)	[121]

Abbreviations: ^1^ Gelatin methacryloyl, ^2^ Polyurethane, ^3^ Bone morphogenetic protein-2, ^4^ Poly(lactic-co-glycolic acid), ^5^ Insulin-like growth factor-1|Brain-derived neurotrophic factor, ^6^ Poly(lactic-co-glycolic acid), ^7^ Polyethylene glycol, ^8^ Polyethylene glycol.

**Table 4:** Biocomposites fabricated via AM techniques. Its blending process, fabrication technique, printing condition (temperature (T), flow rate, and fiber diameter (F_d_)) applications, and outcomes.

Polymer	Filler (wt.%)	Other Elements	Blending	Fabrication Technique	T (°C)	Flow Rate (mm/s)	F_d_ (µm)	Application	Outcomes	Ref.
PCL	G (5–7)	-	Melt	Extrusion	90	12	330	Bone TE + Cancer treatment	Compressive modulus (140 MPa) and tensile strength (4.4 MPa) ↓ hADSCs and Saos-2 cell attachment and proliferation	[126]
PCL	G (<0.8)	P1-Latex protein	Melt	Extrusion	90	20	330	Bone TE	↑ hADSCs attachment and proliferation Earlier and more effective osteogenic differentiation	[128]
PCL	G (<0.8)	-	Melt	Extrusion	90	20	330	Bone TE	In vitro: ↑ MC3T3-E1 cell proliferation ↓ Immune response In vivo: Micro-electric stimulation (10 µA) allowed rat calvaria critical size treatment	[124]
PCL	GO (0.1, 0.5)	-	Solvent	Extrusion	100	1	100	Bone TE	↑ Murine preosteoblast cell attachment and proliferation ↑ Expression of bone morphogenic protein-2 and osteopontin (Days 7 and 14)	[129]
PCL	rGO (0.5)	-	Solvent	Extrusion	100	1.4	325	TE	~185% ↑ in compressive strength ~150% ↑ in stiffness ↑ hADSCs growth and viability	[130]
PLA	GO (5)	PU	Solvent	FDM	210	20	400	TE	90 °C ↑ in degradation temperature ~167% ↑ in compressive strength ~75.7% ↑ in tensile modulus ↑ Mouse embryonic fibroblast proliferation Cytotoxicity was not verified	[101]
PLA	GO (0.3)	-	Solvent	FDM	-	-	100, 200	Bone TE	↑ Hydrophilicity (WCA ↓ to ~60°) 70 °C ↑ in degradation temperature 30% ↑ in Young modulus ↑ Osteosarcoma cell proliferation	[131]
PLA	GNP (14)	Fe_2_O_3_	Solvent	FFF ^1^	215	60	480	Bone TE	~83% ↑ in bioactivity ~37.5% ↑ in stiffness	[132]
PLA	GNP (2)	L-arg ^2^	Solvent	FDM	180	50	400 Bone	TE	43.6% ↑ in tensile strength 28.5% ↑ in flexural strength 60 °C ↑ in degradation temperature 7% ↑ in remaining residual weight Cytotoxicity was not verified	[123]

Abbreviations: ^1^ Fused filament fabrication, ^2^ L-Arginine.

## Data Availability

Not applicable.
